# A case series of legionella pneumonia in the niagara region, canada

**DOI:** 10.1186/2197-425X-3-S1-A352

**Published:** 2015-10-01

**Authors:** SM Cargnelli, J Powis, JLY Tsang

**Affiliations:** Michael G. Degroote School of Medicine, McMaster University, Niagara Region, Canada; Niagara Health System, Medicine, St. Catharines, Canada; University of Toronto, Medicine, Toronto, Canada; Toronto East General Hospital, Toronto, Canada; McMaster University, Mediicine, Hamilton, Canada; Niagara Health System, Medicine, Niagara Region, Canada

## Introduction

Legionella pneumonia (LP), caused by *Legionella pneumophila,* was first recognized in 1976. Symptoms of LP include fatigue, myalgia, fever, headache, confusion, diarrhea, nausea, cough, hyponatremia and hypophosphatemia. LP accounts for 2-15% of all hospitalized community-acquired pneumonia (CAP) and carries a mortality rate of 15-25%.

## Objectives

The objectives of this study are to

1) describe hospitalized LP cases in the Niagara Region, Ontario, Canada in 2013, and

2) to validate the Winthrop University Hospital (WUH) point score on our patient cohort.

## Methods

We conducted a retrospective cohort study on all hospitalized LP cases in the Niagara Region, Ontario, Canada in 2013. Local research ethics approval was obtained. Patient data was extracted from electronic and paper medical records.

## Results

Public Health Ontario has confirmed 17 cases of Legionellosis from June to December of 2013. 14 cases were hospitalized with LP. 13 cases of LP were confirmed with urinary antigen test. 1 case was confirmed by polymerase chain reaction of bronchial washing. The median age was 57 years, 8 patients (57%) were men; 3 (21%) had a history of ischemic heart disease, 9 (64%) had hypertension, 5 (36%) had diabetes, 2 (14%) had chronic obstructive pulmonary disease, 4 (29%) had chronic renal insufficiency, 1 (7%) had malignancy and 3 (21%) were immunocompromised. 10 (71%) were cigarette smoker and 3 (21%) were cannabis users. The most common symptoms were fever (71%; mean temperature, 38.6°C), change of mental status (50%), productive cough (50%), and unproductive cough (36%). Contrary to the literatures, only a small proportion of patients reported diarrhea (29%), abdominal pain (29%), nausea and vomiting (21%), headache (14%), fatigue (29%), and myalgia (21%). Patients were more likely to present with unilateral airspace disease (64%) than bilateral airspace disease (36%) on chest xray. The median sodium, phosphate and creatine kinase were 133 mmol/L, 0.84 mmol/L, and 440 u/L respectively. The severity of illness was high in this cohort reflected by a median Pneumonia Severity Index of 119. 8 (57%) were considered likely whereas 6 (43%) were considered very likely to have LP based on the WUH point score. The median presenting WUH point score was 15. 4 (29%) had delayed antibiotics therapy (> 24 hours). 6 (43%) received fluoroquinolone, and 10 (71%) received macrolide. 9 (64%) required endotracheal intubation with a median length of ventilation of 10 days. 8 (57%) required vasopressive support and 5 (36%) required renal replacement therapy. 3 (21%) died. 11 (79%) required intensive care unit (ICU) admission with a median ICU length of stay (LOS) of 14 days. The median hospital LOS of all patients was 11 days.

## Conclusions

LP is a serious form of CAP that carries a high mortality rate despite appropriate antibiotic therapy. Symptoms of LP were not always consistent with the literatures but the WUH point score was helpful.

**Figure 1:**
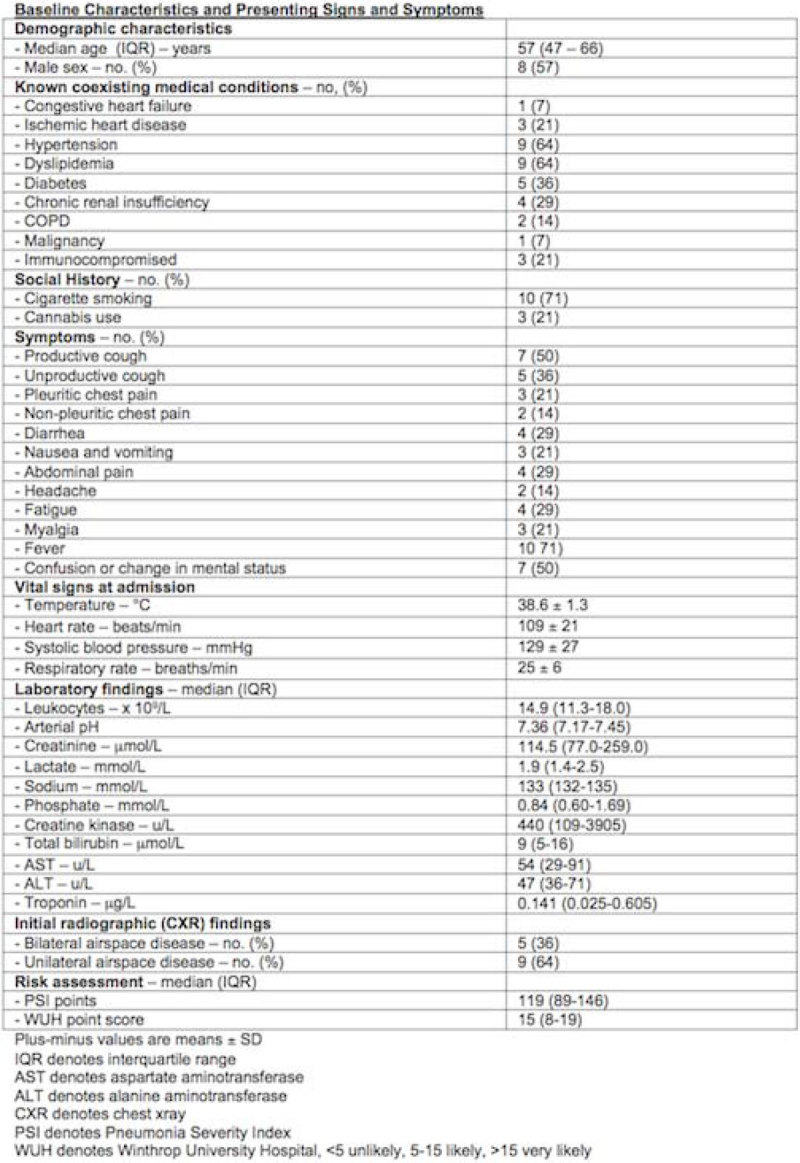
**Baseline and Presenting Characteristics.**

**Figure 2:**
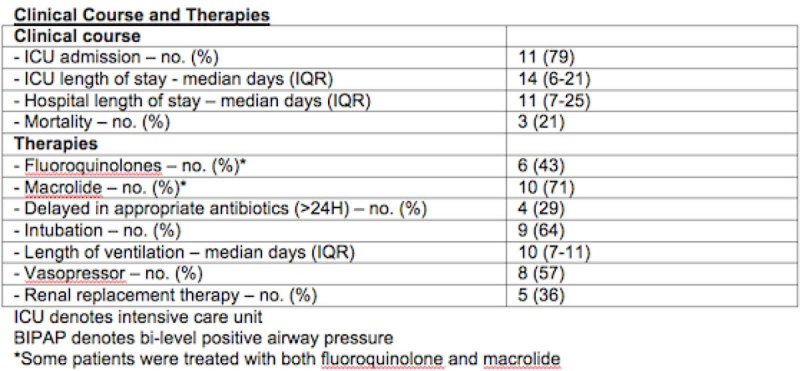
**Clinical Course and Therapies.**

